# A Mysterious Tale of Isolated Right Ventricular Takotsubu Cardiomyopathy

**DOI:** 10.7759/cureus.15742

**Published:** 2021-06-18

**Authors:** Amr Mohamed

**Affiliations:** 1 Internal Medicine, Rochester Regional Health, Rochester, USA

**Keywords:** cardiac arrest, takotsubu cardiomyopathy, right ventricular dysfunction, right sided cardiogenic shock, acute renal failure

## Abstract

Stress cardiomyopathy is typically a diagnosis of exclusion after appropriate rule out of other etiologies of ventricular dysfunction. It most commonly affects the left ventricle (LV). It rarely affects the right ventricle (RV) alone. Here we present a rare clinical dilemma in the setting of cardiac arrest, which occurred in the setting of a stressful event where the final diagnosis ended up being right ventricular takotsubu cardiomyopathy.

## Introduction

Stress cardiomyopathy usually involves the left ventricle (LV) and is typically a diagnosis of exclusion [1]. When we see a shocked patient with isolated right ventricular (RV) dysfunction in the ICU environment, we usually think about acute pulmonary embolism; we never think about stress cardiomyopathy as a separate entity when the dysfunction is isolated to the RV. In this case, we will present a mysterious presentation for isolated right ventricular takotsubu cardiomyopathy.

## Case presentation

A 72-year-old female with a history of asthma was admitted to the hospital after experiencing pulseless electrical activity (PEA) cardiac arrest. It all started after a stressful family fight; 30 minutes later, the patient passed out and had no pulse. Emergency medical service (EMS) arrived with the reported downtime of 10 minutes, followed by return of spontaneous circulation (ROSC).

**Figure 1:**
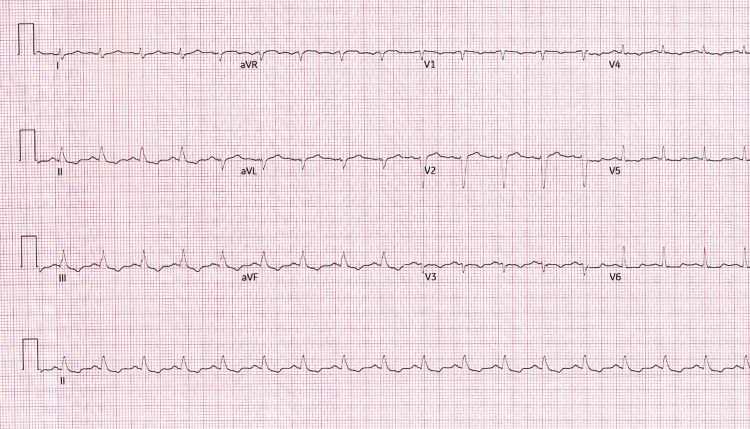
12 lead surface EKG showing sinus tachycardia with non-specific ST segment and T wave changes.

On arrival at the hospital, she was shocked, requiring norepinephrine. EKG showed non-specific ST segment and T wave changes, as shown in Figure 1. Echocardiogram showed right ventricle (RV) dilatation with a D-shaped septum, which raised the suspicion for massive pulmonary embolism despite that she was oxygenating well on the ventilator. Unfortunately, CT scan for pulmonary embolism (CT-PE) could not be done due to acute kidney injury (AKI) with creatinine up to 5 mg/dl.

She was started on therapeutic heparin and was off vasopressors within 48 hours. A ventilation-perfusion scan was done 24 hours later, which showed intermediate probability, so it did not prove or rule out the possibility of pulmonary embolism. Duplex venous lower extremities were negative. Repeat echocardiogram after 72 hours showed that the right ventricular dysfunction had resolved. After the AKI resolved, CT PE was done, which was negative for pulmonary embolism. This was followed by a coronary angiogram which was non-revealing.

Now given reversible RV dysfunction in the setting of negative CT PE and a clean coronary angiogram, a diagnosis of RV takotsubu cardiomyopathy was presumed.

## Discussion

Stress cardiomyopathy is transient regional systolic dysfunction with poorly understood pathophysiology [1]. The most clinically useful criteria that we use are the Mayo Clinic diagnostic criteria, all four of which are required for the diagnosis [1,2]:

1. Transient left ventricular (LV) systolic dysfunction.

2. Absence of obstructive coronary disease.

3. New electrocardiographic abnormalities or modest elevation in cardiac troponin.

4. Absence of pheochromocytoma or myocarditis.

The applicability of the mayo clinical diagnostic criteria to our case is not very practical as with isolated RV dysfunction, ruling out pulmonary embolism is critical, particularly in the setting of cardiac arrest.

It is imperative to delineate that suspicion of stress cardiomyopathy should not delay managing the case as an acute coronary syndrome or pulmonary embolism as stress cardiomyopathy is typically a diagnosis of exclusion. The most common variant of takotsubu cardiomyopathy is apical left ventricular; other rare variants include mid-ventricular, basal, focal, and global variants [3]. Isolated RV takotsubu is not frequently described in the literature.

The management of takotsubu cardiomyopathy is purely supportive, with particular emphasis on the pathophysiology of the shock state. For example, when stress cardiomyopathy is in the left ventricle, sometimes the mechanism of the shock is left ventricular outflow tract obstruction attributable to hypercontractile basal segments rather than related to the poor cardiac pump [3], while with RV takotsubu, the mechanism is poor RV cardiac output. With a long-term follow-up of takotsubo cardiomyopathy patients, all-cause mortality was 5.6%, and the reported major adverse cardiac and cerebrovascular events were 9.9% [4].

## Conclusions

The case serves as the first report for cardiac arrest as a presentation for isolated RV takotsubu cardiomyopathy. Till now, isolated RV takotsubu is not identified as a separate entity or subtype of stress cardiomyopathy but instead as a concomitant to LV dysfunction in some cases. Also, CT pulmonary angiogram is not a prerequisite in the guidelines for diagnosing takotsubu cardiomyopathies. Both of the above will need to be addressed in updated guidelines to make it easier for clinicians to diagnose this condition.
